# Edge-to-edge repair of the systolic anterior motion of mitral valve and cardiac myectomy of the abnormally positioned papillary muscles in an infant with *COL4A1* mutation

**DOI:** 10.1093/jscr/rjab240

**Published:** 2021-06-25

**Authors:** Satoshi Miyairi, Daisuke Takeyoshi, Natsuya Ishikawa, Hiroyuki Kamiya

**Affiliations:** Department of Cardiac Surgery, Asahikawa Medical University, Asahikawa 078-8510, Japan

## Abstract

Surgical treatment is challenging in pediatric patients with left ventricular outflow tract (LVOT) stenosis (LVOTS). We herein present the case of a 2-year-old male patient with porencephaly who was diagnosed with LVOTS accompanied by moderate mitral valve regurgitation (MR) with systolic anterior motion (SAM). Edge-to-edge mitral valve reconstruction and myectomy of the abnormal cardiac muscle were performed, with an uneventful postoperative course. LVOT myectomy and edge-to-edge mitral valve repair may be considered as a safe and acceptable approach with good clinical outcomes in pediatric patients with LVOTS accompanied by MR with SAM.

## INTRODUCTION

Left ventricular outflow tract stenosis (LVOTS), a major cause of sudden death in young adults, is usually due to hypertrophic cardiomyopathy in pediatric patients, who can sometimes present with ventricular dysfunction or arrhythmia [1]. LVOTS often appears with mitral regurgitation (MR) and systolic anterior motion (SAM), with or without extensive hypertrophy of the interventricular septum, and repair of LVOTS remains challenging because of the variety in presentation and patient age.

In this report, we present the case of a 2-year-old male patient with MR and SAM caused by abnormally positioned papillary muscles, in whom SAM led to severe LVOTS. The patient was successfully treated with edge-to-edge mitral valve reconstruction and myectomy of the abnormal papillary muscles.

## CASE REPORT

**Figure 1:**
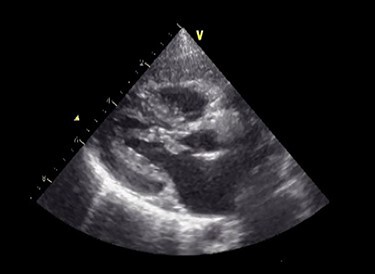
Preoperative echocardiography. The abnormal muscle band in left ventricle and severe SAM of the mitral valve are observed. The anterior leaflet of mitral valve obstructs the LVOT in the systolic phase.

A male infant born at 34-week gestation with 1531-g birth weight was diagnosed with porencephaly based on fetal examination. The patient had persistent hemolytic anemia and jaundice and was diagnosed with a mutation in collagen type IV alpha 1 chain (*COL4A1*) by postnatal genetic analysis. The details of metabolic and muscular complications of the patient were described in a previous report [2]. The patient underwent surgery for ventriculoperitoneal shunt placement for hydrocephalus and seizure at the age of 3 months and was hospitalized several times for recurrent pneumonia until the age of 2 years, at which time he was diagnosed with LVOTS and moderate MR. The patient was treated with β-blockers for 6 months, but LVOTS and MR gradually worsened and SAM of mitral valve was detected at the age of 2 years and 8 months. At the time, the peak pressure gradient of left ventricular outflow tract (LVOT) was 120 mmHg and the MR was severe (Fig. 1 and Video 1). Surgery was planned for septal myectomy for LVOT with mitral valve repair.

**Figure 2:**
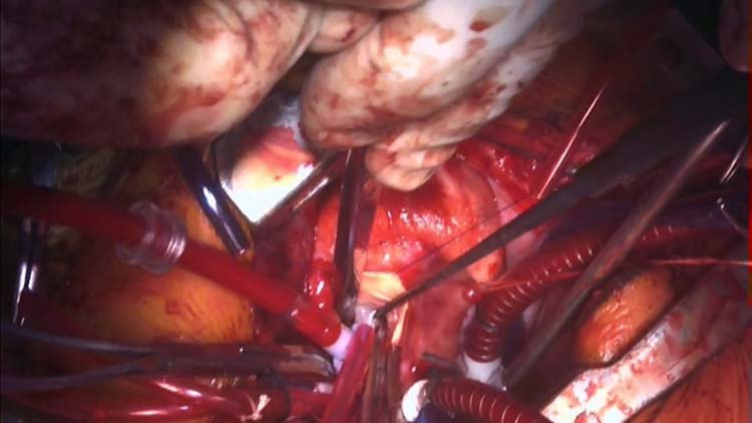
Intraoperative findings. The abnormal muscle band is cut by scalpel, and edge-to-edge repair of the mitral valve is performed.

**Figure 3:**
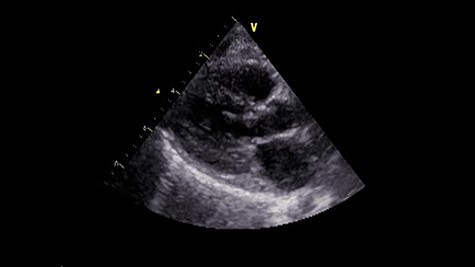
Postoperative echocardiography. The abnormal muscle band has disappeared, and the SAM of mitral valve is well controlled.

Under general anesthesia, cardiopulmonary bypass was established by ascending aortic and bicaval cannulation. After transverse aortotomy, LVOT was exposed through the aortic valve by retracting the aortic wall. The cardiac muscle hypertrophy with abnormal bundle-like cardiac muscle was observed in the LVOT, including the septal area. The hypertrophied area was scooped up by a nerve hook and cut by scalpel (Fig. 2 and Video 2). Next, the left atrium was incised and the mitral valve was exposed. The edge-to-edge technique with a suture of the A2–P2 area was performed for mitral valve reconstruction. Postoperative recovery was uneventful, and echocardiography revealed improved LVOTS with 1.7-m/s peak velocity and no MR or mitral stenosis (Fig. 3 and Video 3).

## DISCUSSION

Surgical intervention remains a challenge in infants with LVOTS. In a study of 23 pediatric patients with LVOTS undergoing septal myectomy, including 12 patients younger than 1 year of age, Schleihaul *et al*. [1] reported that the long-term outcomes were favorable between the infants and older patients. Another study reported that septal myectomy did not increase myocardial extracellular volume fraction and did not affect conduction [2].

Regarding mitral valve repair in patients with LVOTS, Salah *et al*. reported that 95% of the pediatric patients with LVOTS who underwent septal myectomy had concurrent SAM and MR [3]. In that study, 12 of the 17 cases underwent mitral repair, but the details of mitral valve repair were not provided. The edge-to-edge repair of mitral valve in infants is uncommon. Vricella *et al.* [4] reported that surgical MR correction using ring annuloplasty and edge-to-edge repair was associated with good mid-/long-term clinical outcomes in pediatric patients with connective tissue diseases without SAM or MR. These studies suggest edge-to-edge mitral repair and septal myectomy in infants as good surgical options; however, most of the reported cases were adolescent patients. Yoshizawa *et al*. [5] reported six infants with idiopathic acute MR who underwent edge-to-edge mitral valve repair. The authors used Kay’s annuloplasty and artificial chordae in some cases and reported no early or late deaths or reoperation.

One benefit of the edge-to-edge mitral valve repair is the simple technique, which reduces surgical duration, with good outcomes. In this case, edge-to-edge mitral valve repair was done for SAM. Since this case had gene mutation and neurological abnormality, we need to avoid risks from second run of cardiopulmonary bypass or prolonged time bypass. In complicated cases or those requiring simultaneous cardiac surgical procedures, the edge-to-edge repair may be a favorable option, especially in infant or neonatal cases.

In summary, the present pediatric patient with LVOTS accompanied by MR with SAM underwent resection of the abnormal cardiac muscle and edge-to-edge mitral valve repair, with an uneventful postoperative course, resolution of MR and mild LVOTS. The current case illustrates surgical left ventricular septal myectomy with edge-to-edge mitral valve repair as a favorable option in infant patients with LVOTS and MR with SAM.

## Supplementary Material

Video_1_rjab240

Video_2_rjab240

Video_3_rjab240
